# Abundance of sulfur‐degrading bacteria in a benthic bacterial community of shallow sea sediment in the off‐Terengganu coast of the South China Sea

**DOI:** 10.1002/mbo3.380

**Published:** 2016-06-03

**Authors:** Zahar Marziah, Akbariah Mahdzir, Md. Nor Musa, Abu Bakar Jaafar, Azran Azhim, Hirofumi Hara

**Affiliations:** ^1^ Malaysia‐Japan International Institute of TechnologyUniversiti Teknologi MalaysiaJalan Sultan Yahya PetraKuala Lumpur54100Malaysia; ^2^ Ocean Thermal Energy Centre (OTEC‐KL)Universiti Teknologi MalaysiaJalan Sultan Yahya PetraKuala Lumpur54100Malaysia; ^3^ Perdana School of ScienceTechnology and Innovation PolicyUniversiti Teknologi MalaysiaJalan Sultan Yahya PetraKuala Lumpur54100Malaysia; ^4^ Kuliyyah of ScienceInternational Islamic University MalaysiaKuantan25200Malaysia

**Keywords:** Biodiversity, environmental microbiology, hydrocarbon degradation, marine metagenome

## Abstract

This study for the first time provides insight into the bacterial community in the benthic region of the Off‐Terengganu Coastline, which is considered to be anthropogenically polluted due to heavy fishing vessel commotion. Subsurface bacteria were randomly collected from two locations at different depths and were examined using the 16S rDNA V3‐V4 marker gene on the Illumina^™^ Miseq platform. In addition, the physiochemical parameters of the sediment were also measured. Surprisingly, the results show a high diversity of sulfur‐oxidizing bacteria in the surveyed area, where *Sulfurovum* sp. was identified to predominate the overall bacterial community. The physiochemical parameters reveal insufficient evidence of hydrothermal vents in the surveyed area. However, there are traces of hydrocarbon pollutants such as gasoline, diesel, and mineral oil in this area. It is assumed that sediment accumulation in the lee of breakwater plays an important role in trapping the runoff from the nearby harbor, which includes oil spills. Based on the common knowledge, *Sulvurofum* sp. is a native bacterium that exists in deep hydrothermal vents and volcanic territories. Although the reason for the abundance of *Sulfurovum* sp. in the surveyed area is still unclear, there is a possibility that metabolic adaptation plays an important role in regulating hydrocarbon pollutants for survival. The work presented in this paper therefore has profound implications for future studies on *Sulfurovum* sp. versatility. However, future research is needed to strengthen the findings of this study and to provide a better evidence regarding the metabolic response of this bacterium toward hydrocarbon pollutants.

## Introduction

Marine life has existed in oceans since almost 3.5 billion years when microbes were the only form of life in two‐thirds of the planet's existence. Although their diversity and metabolic contribution in marine microbiology remains indecisive (Munn 2011), it is widely assumed that marine microbes play a vital role in regulating marine ecological processes, which include the biogeochemical cycles (Danovaro et al. 2015). In recent years, marine microbial research is inclined to investigate the microbial community profiles in order to expand the species identification and variance in different environments (Zhu et al. 2013; Bokulich et al.*,* 2014; Wang et al., 2015b). In recent years, marine microbial deviation and its mechanism are mainly investigated in local communities and may usually include studies on physiochemical parameters to justify the microbial metabolic activities (Van der Gucht et al. 2007 and Wang et al. 2015a, 2015b).

**Figure 1:**
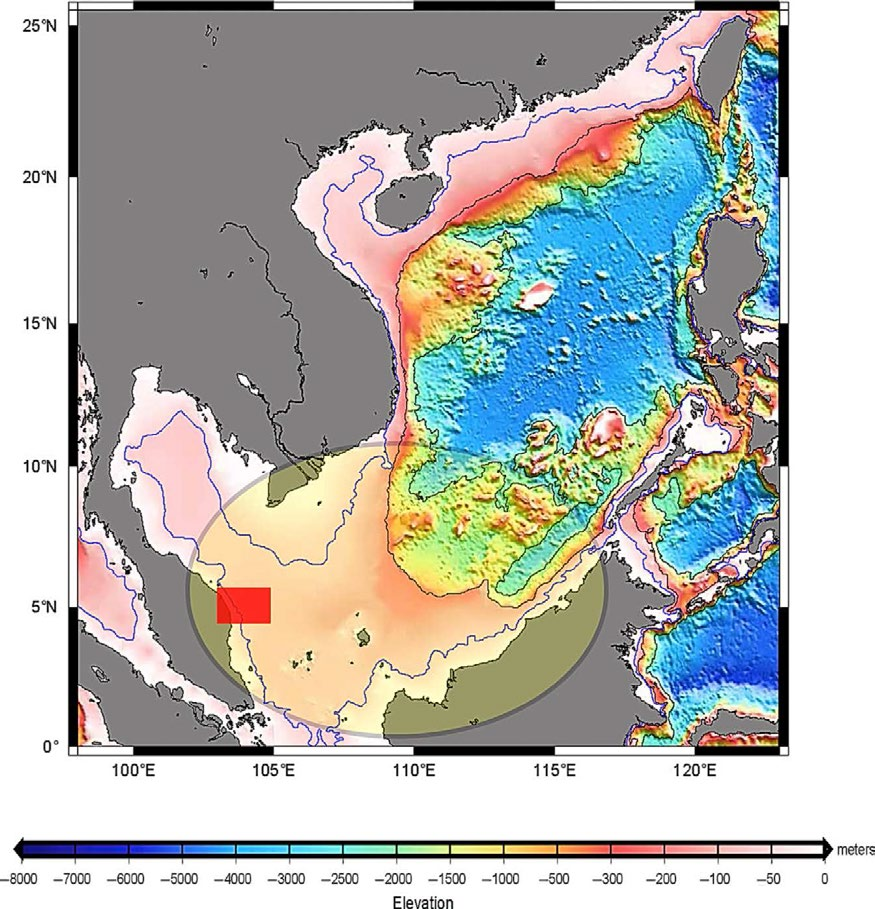
Illustration of South China Sea seafloor bathymetry. The Southern part of the South China Sea is marked in an ellipse. The area bounded by the rectangle indicates the region of interest; Terengganu State in Peninsular Malaysia (5°N 103°E) (Image courtesy of Liu and Dittert 2010).

In this study, the region of interest was located in the Southern South China Sea (SSCS) (refer to Fig. 1). Geographically, the SSCS has a shallow, ±50 m neritic epipelagic seabed and, presumably has effective photosynthesis, which contributes to a high coral distribution globally (Taylor and Hayes 1983; Morton and Blackmore 2001; Wang et al. 2007a,b). In addition, the SSCS is signified as the heart that connects Eurasia with the Americas, as the largest shipping port in the world is located here (Fan et al., 2016). Furthermore, the SSCS clenches huge reserves of Tapis‐grade crude oil beneath its seabed (Ismail et al. 2015).

**Figure 2:**
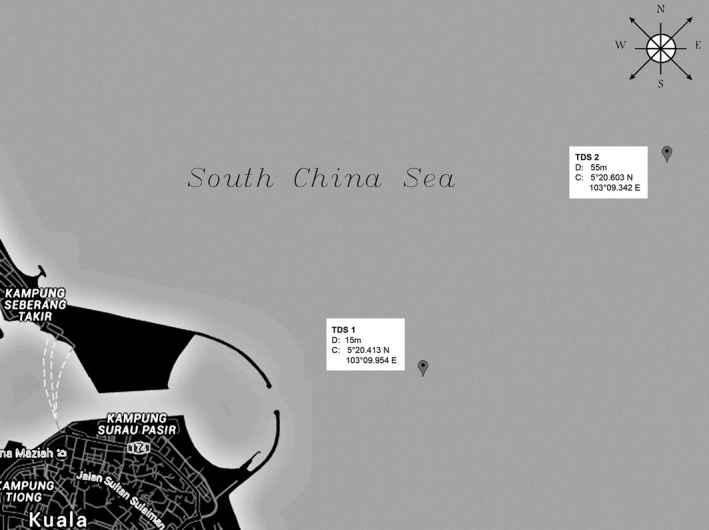
Illustration of sampling points and breakwater structure located in the Off‐Terengganu coastline.

This study was conducted off the Terengganu coastline area (5°20'N, 103°09'E) as a part of bacterial diversity research to contribute toward the understanding of Malaysian waters, which is also a part of the SSCS region. In addition to the neritic epipelagic bed, this area has an immense collection of tropical marine life forms (Arai 2015), besides being surrounded by a high‐quality crude oil cluster (Ismail et al. 2015). One prominent feature that represents this particular Off‐Terengganu coastline is the presence of a stern‐curve breakwater (refer to Fig. 2) that was constructed to protect the Kuala Terengganu jetty and the nearby estuaries.

A few conditions might influence the outcomes of the bacterial phylogenetic profile in the Off‐Terengganu coastline. In a recent study, high values of biochemical oxygen demand (BOD), chemical oxygen demand (COD), total suspended solids (TSS), and ammonical nitrogen (AN) were recorded in the Kuala Terengganu river, which is directly connected to the Off‐Terengganu coastline (Suratman et al. 2015). Another study specifies that anthropogenic sources such as municipal waste, surface runoff, agricultural runoff, organic pollution, and urban storm runoff have polluted this location (Kamaruddin et al. 2015). Given the context of possible sediment amiability toward the anthropogenic pollutant potency in the surveyed areas, the bacterial phylogenetic profile might not represent an abundance of the native marine bacterial community. Instead, it may actually illustrate a unique bacterial community with the capability to utilize inorganic compounds such as sulfur as its food sources, or, perhaps, a water‐borne bacterium that poses a threat in causing disease to the marine community and to humans.

The aim of this study is mainly to create a steadfast foundation of the marine bacterial community in the SSCS region – specifically in Malaysian seawater – since no published phylogenetic profile has been conducted in the surveyed areas. This study also comprises of several physico‐geochemical analyses to investigate the sediment constituents.

## Materials and Methods

Based on Table 1, sampling activity was conducted on 30th November, 2014 using the Smith McIntyre grab sampler (0.1 m^2^ of grab size) at two points (Labeled as “TDS1” and “TDS2”) in a north‐eastern direction. This sampling method was based on the methodology recommended by Holme and McIntyre (1984) where all samples must be handled carefully, kept in a double‐layered polyethylene bag, and stored in a freezer at −25°C until further analysis. No specific permits were required for the described sampling because it did not involve any endangered species nor did it occur within a designated marine‐protected area or private reserved park.

**Table 1:** Location information for sampling sites

Sampling Sites	Longitude (E)	Latitude (N)	Time of sampling (hours)	Depth (m)	Approximate distance initial points^a^ (km)
TSD1	103°09.954'E	5°20.413'N	09:45	±15	4.01
TSD2	103°09.342'E	5°20.603'N	13:50	±55	8.27

a The initial points located in the Pulau Duyong Harbour, Kuala Terengganu. Approximately 4.01 km from the first sampling point.

### Isolation and bacterial characterization

Genomic DNA was extracted from 1 g of sediment using the PowerSoil®DNA Isolation Kit (MO BIO, Carlsbad, CA) according to the manufacturer's protocols. The extracted DNA product was assessed for its integrity and concentration by a standard agarose gel analysis. Qubit® 2.0 DNA Kit (Invitrogen by Thermo‐Scientific Inc. Waltham, MA) was used to quantify the precise DNA product to ensure sufficient DNA amounts for the Polymerase Chain Reaction (PCR). The primer used for PCR was a universal primer for 16SrDNA that targeted the V3 region: ‐341F (5′ CCTACGGGN GGCWGCAG 3′) and 805R (5′ GACTACHVGGGTATCTAATCC 3′). The amplified product was then analyzed by agarose gel electrophoresis and recovered with the Sangon agarose recovery kit (Sangon Biotech Co., Ltd., Shanghai, China). Subsequently, the recovered DNA products were quantified and mixed to 1:1 ratio based on DNA concentration determined by Qubit® 2.0 Fluorometer (Invitrogen by Thermo‐Scientific Inc.). Pyrosequencing analysis was conducted on the Illumina® Miseq platform (Illumina Inc., San Diego, CA) at Sangon Biotech Co., Ltd..

### DNA sequence analysis

A total of 37,363 sequences that span the 16S rDNA V3‐V4 hypervariable region were identified and filtered using the Illumina Miseq^™^ platform (Illumina Inc.,). Random sequences, ambiguous residues, and sequence lengths of than 150 bp were eliminated. Quality control (QC) for the raw sequences was performed with PRINSEQ‐lite 0.19.5 to truncate the low‐quality data and improve the merge ratio for subsequent sequences. Using Flash v1.2.7 (University of Maryland, MD, USA), the raw sequence fragment was merged in a dual terminal to form a single primer. Subsequently, short, low‐complexity, and low‐quality primer fragments were eliminated by PRINSEQ‐lite 0.19.5 software (Soundforge Media, La Jolla, CA). Correction of sequencing errors was performed with precluster software and was integrated by the Mothur software (Michigan, MI, USA). Subsequently, chimeras and extraterritorial sequences of the target area were removed with the Uchime software using SILVA data as the template. By the time the QC ended, primer length was successfully aligned between 400–500 bp, with an average of 450 bp. All V3 and V4 optimized sequence reads were determined by RDP classifier 16S (Wang et al. 2007a,b) and Silva 16S (Quast et al. 2013).

### Diversity and statistical analysis

The sequence parameter for similarity and operational taxonomic unit (OTU) was set to 97%, which is close to genus probability. For the first step, OTU clustering was performed using UCLUST v.1.1.579 to select the longest reads from the clean sequence as seed sequences (Edgar 2010). In the second step – a sequence with similarity to the seed sequence within the threshold range – was then selected. Finally, all the sequences obtained from the first and second steps were classified into one OTU category. All three steps of the above process were repeated until all the sequences were successfully classified. The taxonomic unit was classified with the RDP classifier based on Bergey's taxonomy using Bayesian assignment calculation to calculate the probability of each sequence being assigned to the rank on the genus level. One representative sequence with the highest OTU abundance was automatically distinguished by the RDP classifier to categorize the species, with the default value of taxonomy threshold being 0.8/0.5. A cluster of multiple sequences based on the distance between sequences, OTU classifications, and the similarity of the sequence threshold value was determined by the Mothur^™^ software. Subsequently, all sequence clusters were calculated based on the *α*‐diversity index analysis (based on Richness index, Shannon index, ACE index, Chao1 index). The rarefaction curve value and graph were generated based on 97% of the sequence similarity threshold on every species, genus, and family level analyzed. *β*‐diversity index analysis was excluded from the diversity study due to data deficiency (which requires at least three samples to generate a satisfactory *β*‐diversity index).

### Physico‐chemical analysis

#### In situ water quality analysis

The Hydrolab Multiparameter Sonde DS5X was used to evaluate the in situ water quality, with seven parameters analyzed: temperature, pH, specific conductivity, salinity, total dissolve solid (TDS), turbidity, and luminescent dissolved oxygen (LDO). The multiparameter probe was cleaned and calibrated prior to each sampling session. Eleven to 12 readings for each parameter were obtained in a single point where every output was directly linked (by GPS) and recorded into the Aqualab Hydras 3 LT Software for Microsoft^®^ Windows 7. Statistical analysis was performed with SPSS 16.0 (SPSS Inc. Chicago, U.S) for Microsoft^®^ Windows 7. The results were interpreted based on Pearson correlation with *P* ≤ 0.05 and *P* ≤ 0.01 being considered as significant.

#### CHNS elemental analysis

Rapid identification of carbon (C), hydrogen (H), nitrogen (N), and sulfur (S) in the sedimentary sample was performed using the Vario MACRO^™^ cube CHNS is acronym that is combined from Carbon (C), Hydrogen (H), Nitrogen (N) and, Sulfur (S). (Elementary, Deutschland). The sediment samples were air‐dried in a 50°C oven and then ground, sieved (<2 mm), and homogenized according to the ISO 2004 protocol. Sulfur determination was conducted according to the ISO 2005 protocol.

#### Oil and grease analysis

Oil and grease (O&G) determination was conducted using a partition‐gravimetric method. Specifically, the Hexane Extractable Method – USEPA 1664 (EPA 1999) was used. The oil and grease in the sediment were extracted from water and then attached to *n*‐Hexane solvent. The solvent was allowed to evaporate slightly before transferring it to a preweighed culture tube. The solvent was further evaporated completely until dry. The culture tubes were then weighed again (EPA 1999 and Bucci et al. 2015).

#### TPH analysis

Total petroleum hydrocarbon (TPH) was measured based on the USEPA 8015B test method (EPA 2000). A quantity of 10 g of chilled fresh sediment was transferred into vials with a solid cap and a Teflon septum. A quantity of 20 mL of *n*‐Pentane solution was added to the same vial and mixed homogenously by centrifugation for 15 min. The mixture was allowed to settle for 1 hour at room temperature and then considered ready for gas chromatography with flame ionization detector (GC‐FID) analysis. Each sample mixture was passed through the Agilent J&W Capillary (DB‐5 30 m × 0.25 mm × 0.25 *μ*m) into the Agilent 7890A GC‐FID with a carrier gas (Helium) flow rate of 40 cm/sec. Internal quality control was performed by considering ±5% as the acceptance criteria.

## Results

A total of 15,268 cleaned effective sequences based on 16S rDNA V3‐V4 were grouped into 2,156 unique OTUs (Operational Taxonomic Unit), where one OTU denotes a sequence with an identity value equal to or higher than 97% (Zhu et al. 2013; Wang et al. 2015a, 2015b). Based on the *α*‐diversity index report in Table 2, TSD1 demonstrated higher species richness compared to TSD2 by 70%. This was most likely contributed by the lush sea grass vegetation in the TSD1 area that enhanced organic matter accretion in the sediment (Baden et al. 2010; Jankowska et al. 2015). The previously abundant marine bacteria gradually decline with increasing depth of the seawater. This is assumed to be due to the reduced seagrass vegetation at these depths (García‐Martínez et al. 2008; Jankowska et al. 2015). In general, the accumulation of organic and inorganic matters in seagrass allows marine bacteria to regulate nitrogen and phosphorous cycling, and thereby, maintain seagrass productivity in seawater (Donnelly and Herbert 1998).

**Table 2:** The list of alpha diversity index cumulative results for TSD1 and TSD2

Sample ID	Seq. num	OTU num	Shannon index	ACE index	Chao1 index	Coverage index
TSD1	8210	1496	3.8097	8407.942456	4711.624	0.865652
TSD2	7058	660	2.0567	5949.750563	2538.676	0.92675

OTU, operational taxonomic unit.

### Phylogenetic profile report

**Figure 3:**
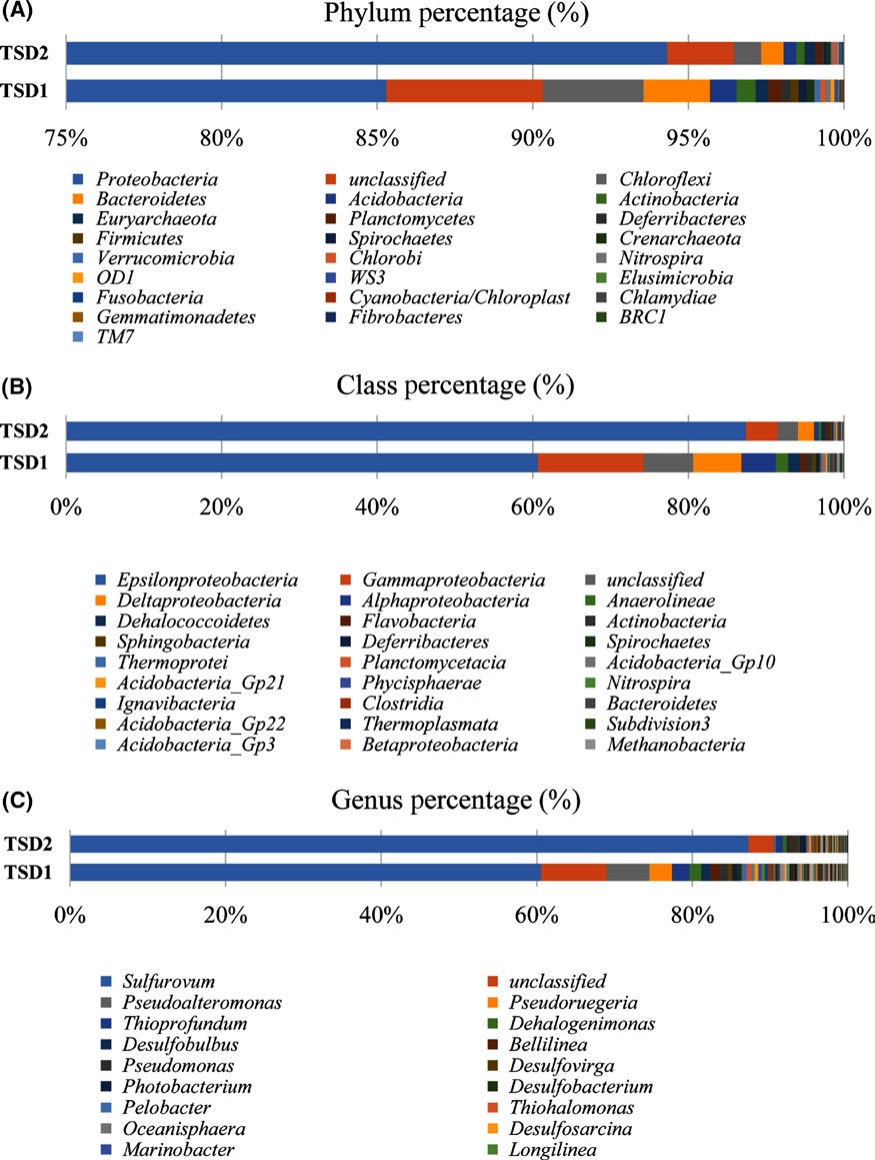
An Illustration of bacterial diversity and phylogenetic distribution based on RDP classifier 16S (Wang et al. 2007a,b) and Silva 16S (Quast et al. 2013). The RDP bar was generated based on operational taxonomic unit with the range of 21–27 phylum levels, 35–51 class levels, 46–62 order levels, 76–108 family levels, and 134–217 of genus levels identified in two sampling locations, respectively.

The bacterial phylogenetic profiles are as depicted in Figure 3. The results indicate that the phylum Proteobacteria tops the overall phylum abundance in the surveyed area with 85% (TSD 1) and 95% (TSD 2). In a general perspective, Epsilonproteobacteria predominates in the bacterial community with 60% (TSD 1) and 88% (TSD2), respectively. This is followed by Gammaproteobacteria with 15% and 5%, Deltaproteobacteria with 5% and 3%, and Alphaproteobacteria with 4% and 1%, respectively. These results also show that an unclassified phylum is the second most abundant phylum detected with 5% and 3%, respectively, followed by the phylum Chloroflexi with 3% and 1%, respectively.

This study reveals that *Sulfurovum* sp. was the only genus that predominantly covered the Epsilonproteobacteria genera in both surveyed areas with 60% (TSD1) and 88% (TSD2). Other than *Sulfurovum* sp., TSD1 also contained several sulfur‐oxidizing genera from Gammaproteobacteria, which include *Thioprofundum, Desulfobulbus, Desulfovirga, Desulfobacterium, Desulfosalsimonas, Sulfurimonas, Sulfuricurvum,* and *Thermodesulfovibrio* (Fig. 3 section C). No inclusion data were obtained to demonstrate the species composition of *Sulfurovum* sp. since the 16S rDNA phylogenetic profile is limited to identifying bacterial genus within the V3–V4 hypervariable region. Therefore, further investigation is required to determine the species composition of *Sulfurovum* sp.

### Physicochemical reports

#### In situ water quality analysis

The bottom of the seawater level in both surveyed areas was measured by in situ water quality analysis (refer to Table 3). The results indicate that the temperature, pH, Sp. conductivity, salinity, TSD, and LDO values in both surveyed areas are significant. However, seawater turbidity in TSD1 indicates an insignificant value (*P* = 1.03573), whereas the value of TSD2 is significant (*P* = 0.0000). Despite a constantly irregular, high turbidity value in TSD1, the probability sediment disruption was caused by wave dissipation in the lee of the breakwater, perhaps resulting in particulate matter retention from water runoff in TSD1 (refer to Fig. 2). A stable sediment layer probably contributed to the zero turbidity value in TSD2 because it is remotely located from the breakwater (Qi and Gao 2015).

**Table 3:** Result of environmental conditions and physio‐geochemical analyses

No	Parameters	TSD1	TSD2
	**Bottom seawater in situ water quality analysis**		
1	Temp (°C)	30.5227 ± 0.00647	30.0683 ± 0.00389
2	pH	7.8200 ± 0.02	7.9325 ± 0.00452
4	Specific conductivity (mS/cm)	60.8091 ± 0.5394	60.700 ± 0.4264
5	Salinity (ppt)	40.8655 ± 0.2734	40.7958 ± 0.3288
6	TDS (g/L)	38.9091 ± 0.3015	38.8917 ± 0.2887
7	LDO (mg/L)	6.6482 ± 00874	6.7717 ± 0.06726
	**Sediment TOC analysis**		
8	TOC ppm	4600	5200
	**Sediment elemental analysis (CHNS)**		
9	Carbon (%)	1.86	1.25
10	Hydrogen (%)	1.017	0.035
11	Nitrogen (%)	0.99	0.58
12	Sulfur (%)	0.916	0.212
	**Sediment oil and grease analysis**		
13	HEM (%)	0.47	0.08
	**Sediment TPH analysis**		
14	C_6_–C_9_ (ppm)	0.05	aND
15	C_10_–C_19_ (ppm)	0.10	0.11
16	C_20_–C_36_ (ppm)	0.22	0.29
17	C_37_–C_44_ (ppm)	aND	aND

a ND, Not detected.

TOC, Total Organic Carbon, HEM, Hexane extractable method; TPH, Total Petroleum Hydrocarbon; LDO, Luminescent dissolved oxygen, TDS, Total dissolve solid.

#### Total organic carbon

The results indicate that the total organic carbon (TOC) sedimentary values would increase with depth. In correspondence to the zero water turbidity value in TSD2, it is assumed that sunlight effectively penetrates into the clear water column. This supports photosynthesis, thus creating a better marine food chain environment and generating high organic matter from cell remains (Bell et al. 2015 and Saraswathy et al. 2015) where it demonstrates high TOC value (Bendtsen et al. 2015). However, there is no concrete evidence that links *Sulfurovum* sp. abundance with the high TOC in both seawater and the sedimentary layer.

#### Elemental (CHNS) analysis

The CHNS analysis was mainly performed to observe the overall elemental composition in the surveyed area. Based on Table 3, all four of the main elemental ratios, including sulfur, were scarcely identified. Further analysis is therefore necessary to investigate sulfur concentration in order to demonstrate a convincing association of the *Sulfurovum* sp. with the sulfur content in the surveyed area.

#### Hexane extracted method and total petroleum analysis

Based on the Hexane Extracted Method (HEM) analysis, the oil and grease fragments were fairly identified to be 0.47% (TSD1) and 0.08% (TSD2). Since HEM assessment showed a promising value, it was necessary to thoroughly quantify the hydrocarbon compounds using TPH analysis (Bucci et al. 2015). The outcome of TPH analysis confirmed the existence of gasoline (C_4_–C_9_), diesel (C_10_–C_19_), and organic oil (C_20_–C_36_) fractions in TSD1 at 0.05 ppm, 0.10 ppm, and 0.22 ppm, respectively. Conversely, TSD2 sediment only traced diesel fraction (C10–C19), and organic oil fraction (C20–C36) at 0.11 ppm and 0.29 ppm, respectively. No asphalt / bitumen fraction (C37–C44) was detected in both samples.

## Discussion

One of the interesting features that signify the off‐Terengganu coastline is the presence of a breakwater structure. This breakwater was built mainly to reduce the wave intensity in the inshore water as part of the coastal defense and as an anchorage protection from the weather and longshore drift effects. However, the dissipation of energy and the formation of relative calm water often result in sediment accretion and salient build‐up in the lee of a breakwater (Van Rijn 2010). Furthermore, excessive rainfall inside the breakwater area would cause a runoff that is eventually trapped within the breakwater (Butt 2013). Our finding suggests that turbidity plays an important role in visual prediction of the water condition. It represents a mixture of sediment and xenobiotic compounds that have accumulated for decades. Most of the time, this occurrence would result in unfavorable consequences toward the marine sediment (Jonsson et al. 2006). For example, a 13.4 km breakwater structure was built along the Long Beach, CA coastline to protect the U.S naval ships during World War II. Although the harbor is no longer in use since 1996, the harmful xenobiotic compounds that remained trapped in the lee of breakwater, made the place inaccessible for recreational purposes (Butt 2013). There is a strong probability that TSD1 is positioned in an active wave dissipation area because of water turbidity. Therefore, the sedimentary layer of TSD1 may have richer particulate matter retention from water runoff as compared to TSD2, which ultimately intensifies the concentration of anthropogenic pollutants in the breakwater opening.

Given the context that the majority of bacterial communities in both sampling points are a sulfur‐degrading group, it is expected that the marine sediment possibly contain a sulfur component. The lack of sulfur ratio in the elemental analysis indicates that there are no hydrothermal vents or volcanic rock structures in both surveyed areas. However, aromatic hydrocarbon, which has high affinity for sediment (McElroy et al. 1989) and mainly contributes to marine coastline pollutants (Mitsch 2010; Suárez‐Suárez et al. 2011), was discovered at both sampling points. Both areas were polluted with gasoline, diesel, and mineral oil (excluding TSD2) – perhaps due to oil spills from fishing vessels and high‐speed boats. Based on the available information, 2216 units of fishing vessels were registered in the surveyed area (Kuala Terengganu district) in the year 2001, with 316 units being outboard‐powered vessels while the rest were inboard‐powered vessels (Information of Fisheries Management in Malaysia 2001). The inboard‐powered motors were mainly fueled by gasoline or diesel while the outboard motors were fueled by gasoline with 0–10% of ethanol blended fuel.

Marine bacteria have played an essential role in regulating the oceanic ecosystem for millions of years by controlling the geochemical processes (He et al. 2009). In the pelagic realm, bacteria are indispensable for two major reasons: they are consumed by other organisms and they degrade the organic matter to sustain ecological systems. Bacteria are thus positioned at both the start and the end of the food chain, where they contribute to the production of particulate foodstuff by converting dissolved organic substrates. They are also responsible for the ultimate breakdown of organic matter that leads to the return of nutrients to the sea (Li and Dickie 2003). Bacteria may be the crucial link or sink between detritus, dissolved organic matter, and higher trophic levels. For these reasons, bacteria occupy a central role in two interconnected environmental issues of global concern, namely the sustenance of harvestable living resources and the mitigation of climate change by sequestration of carbon in the deep ocean (Li and Dickie 2003).

To date, Proteobacteria remains the most abundant environmental microbe phylum that is broadly discovered and cultured. It has been widely detected in various environmental conditions such as in cold seawater regions (Sapp et al. 2010 and Stibal et al. 2015), marine sediment (Zhu et al. 2013; Wang et al. 2015a,b), marine sponges (Schmitt et al. 2012), and organic compounds (Kleinsteuber et al. 2008 and Lin et al. 2014). It is also the predominant phylum identified in both surveyed areas at the Off‐Terengganu coastline. However, certain regions demonstrate the dominance of a different clade of Proteobacteria. For example, the Alphaproteobacteria genera dominate the benthic bacterial community in the Atlantic Ocean at 55.7% Zinger et al. 2011 and in the East China Sea at 20.1% (Wang et al. 2015a, 2015b). Meanwhile, Gammaproteobacteria flourish in the Northern South China Sea (NSCS) at 53.4% (Zhu et al. 2013). The results from this study provide unique findings, where they exhibit the first insight into the abundance of the Epsilonproteobacteria group in a shallow, nonhydrothermal feature coastline. Generally, the Epsilonproteobacteria group is widely known for its ecologically significant bacterial community in deep hydrothermal vents (López‐García et al. 2003 and Zhu et al. 2013). Furthermore, it is also abundant in shallow hydrothermal vents (Sunamura et al. 2004; Takai et al. 2004; Giovannelli et al. 2013; Wang et al. 2015b), sulfite compounds (Nakagawa et al. 2005), and deep sea vent metazoans (López‐García et al. 2003; Goffredi et al. 2004). The abundance of Epsilonproteobacteria in anthropogenic pollutants, however, is still not well‐understood (Inagaki et al. 2004; Takai et al. 2004; Nakagawa et al. 2005) even though few recent studies have discovered their potential to survive in certain organic pollutants (Bolhuis et al. 2014; Lin et al. 2014).

Biological activity in marine sediments is vital because it is partially responsible for the primary marine production and the overall geochemical process (Danovaro et al. 2015). It is assumed that most of the bacterial communities that dwell in coastal sediments have a specific purpose. For instance, 50% of the deposited minerals in the coastline setting are mineralized via sulfate reduction (Jørgensen 1982), which is controlled by sulfur‐degrading bacteria (Suárez‐Suárez et al. 2011).

The outcome of this study describes that *Sulfurovum* sp. is the only genus that covers the entire Epsilonproteobacteria phylogeny profile in both surveyed areas. This genus is characterized by its egg‐like coccoidal shape and is capable of oxidizing sulfur for food and survival. (Inagaki et al. 2004 and Takai et al. 2004). *Sulfurovum* sp. is a gram negative, nonmotile genus that is categorized under sulfur‐oxidizing chemoautotrophic genera and was first isolated from a deep sea hydrothermal vent in Okinawa, Japan (Inagaki et al. 2004). It prefers a moderate temperature of between 20°C to 45°C and a medium salinity (Willey et al. 2008). Although the metabolic properties for the majority of the *Sulfurovum* genus remain indecisive, one of the strains, *Sulfurovum* 42BKTT grew chemolithoautotrophically with elemental sulfur or thiosulfate as the sole electron donor and oxygen (optimum 5 % in gas phase) or nitrate as the electron acceptor. The G + C content of its genomic DNA was 48.0 mol% (Inagaki et al. 2004). In a recent finding, the *Sulfurovum* genus inhibits its growth in fresh water rivers (Hubert et al. 2012), high turbidity waters, and acidic conditions (Bolhuis et al. 2014). Therefore, it is probable that the high turbidity value at TSD1 may likely be the main cause of truncated *Sulfurovum* sp. abundance as compared to TSD2.

Overall, *Sulfurovum* is a predominant species identified in deep hydrothermal vents (Inagaki et al. 2004; Wright et al. 2013; Dahle et al. 2015), shallow hydrothermal vents (Giovannelli et al. 2013), volcanic regions (Wang et al. 2015b), caves, sinkholes, and sulfide compounds (Nakagawa et al. 2005; Handley et al. 2012; Jones et al. 2012). Its metabolic versatility was recently recognized where several studies indicate its role in degrading aromatic hydrocarbons (Paissé et al. 2008; Païssé et al. 2010; Håvelsrud et al. 2011; Lin et al. 2014), such as benzene, phenols, and toluene (Kleinsteuber et al. 2008). Furthermore, *Sulfurovum* sp. together with other sulfur‐oxidizing bacteria has the capability to produce active surfactants (Grabowski et al. 2005; Xiu et al. 2010). It is suggested that the majority of the sulfur‐oxidizing bacteria abundant in oil reservoirs are mainly affected by temperature, mineralization, permeability, and water displacement (Lin et al. 2014) and are stimulated by certain heavy metals effluents such as barium, iron, and manganese, which are discharged from hydrocarbon energy plants (Yeung et al. 2011). In a natural environment, the *Sulfurovum* sp. was discovered in hydrocarbon‐polluted coastal seawater such as at a coal oil point in California, USA (Håvelsrud et al. 2011), Berre lagoon in France (Paissé et al. 2008), and Busan Northport in South Korea (Subha et al. 2014). However, none of the above studies exhibit high *Sulfurovum* sp. abundance in a hydrocarbon pollutant compared to its abundance in this particular report at the Off‐Terengganu coastline.

### Novel findings of this study

This research exhibits an alarming possibility that Off‐Terengganu is vulnerable to the impact of anthropogenic pollution. Moreover, no exact calculations have been made to evaluate the anthropogenic‐prone areas along the Off‐Terengganu coastline and in its surrounding areas. Hypothetically, anthropogenic pollution in a marine ecosystem is dependent on the history of environmental pollution itself. It is believed that the bacterial community adapted to a previous oil spill would then recur faster than in a pristine environment due to its metabolic readiness to utilize the hydrocarbon compounds (Paissé et al. 2008).

The novelty of *Sulforuvum* identification at the Off‐Terengganu coastline is evident because this genus was found out of its native preference such as hydrothermal vents and volcanic regions. Furthermore, this study depicted one of the highest *Sulfurovum* sp. distributions ever reported in a natural environment, showing the broadening versatility of its genus in adapting to a different environmental condition. Based on the biodiversity index, *Sulfurovum* sp. abundance shows increase with depth and requires additional effort to generate better species coverage. In the future, this study will require an extensive *Sulfurovum* composition identification and abiotic analysis such as Polycyclic Aromatic Hydrocarbon (PAH) parameter to investigate the interaction of *Sulfurovum* with carcinogenic and toxic compounds in the environment. This will generate better geochemical information, dispersion scale, community variation, and environmental anthropogenic influence.

### Data repository

The sequence data from this research have been deposited in the NCBI's Sequence Read Archive database (http://www.ncbi.nlm.nih.gov/sra) with the temporary submission ID of (SUB1112034).

## Conflicts of Interest

The authors hereby declare there are no conflicts of interest whatsoever.

## Supporting information


**Figure S1.** Illustration of surface current circulation on the South China Sea in June (left) and December (right). The surface current circulation in June indicates the Southwest monsoon season and December indicates the beginning of the Northeast monsoon season (Image was adapted from Bui et al. 2009).

**Figure S2.** The rarefaction curve plot for TDS1 (Red) and TDS2 (Blue) was depicted based on ACE, Chao1, richness, and Shannon diversity indices. The curves were generated based on 97% similarity threshold.
