# Supplementary figures and images for: Abundance of sulfur‐degrading bacteria in a benthic bacterial community of shallow sea sediment in the off‐Terengganu coast of the South China Sea
Source: Microbiologyopen. 2016 Jun 3;5(6):967–78. doi: 10.1002/mbo3.380 (PMC5221450; doi:10.1002/mbo3.380)

Marziah *et al.* 2015 Figure S1

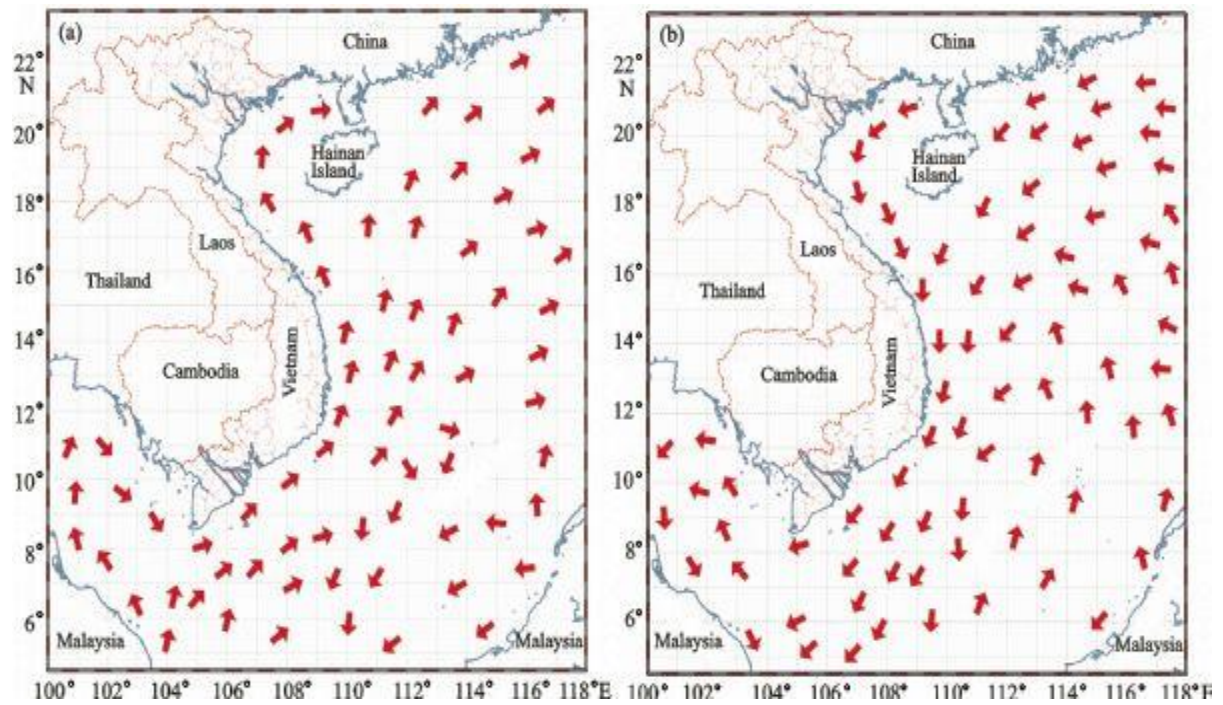

Supplement: Supplementary file 1 — Figure S1. Illustration of surface current circulation on the South China Sea in June (left) and December (right). The surface current circulation in June indicates the Southwest monsoon season and December indicates the beginning of the Northeast monsoon season (Image was adapted from Bui et al. 2009). [file MBO3-5-967-s001.pdf]

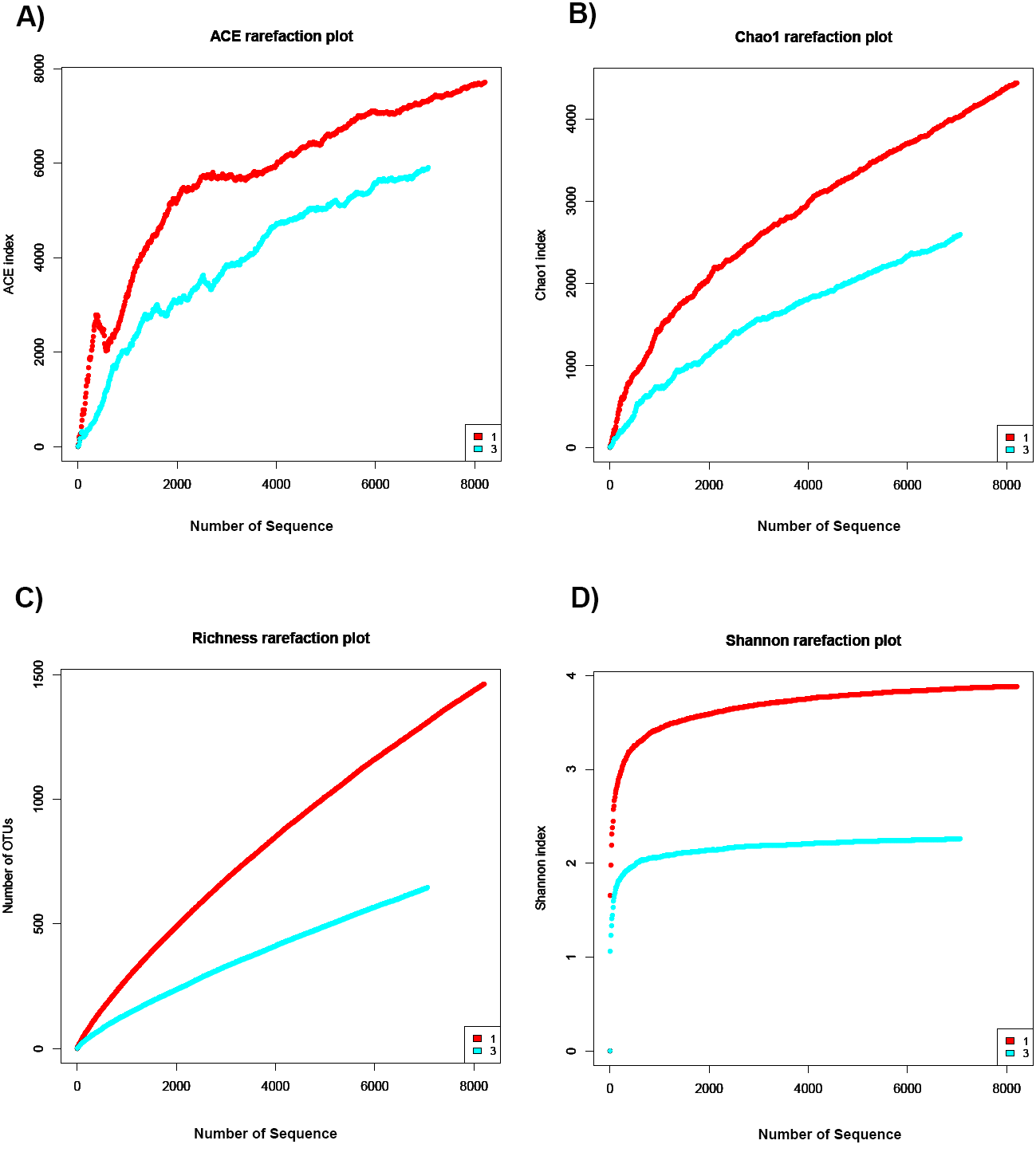

Supplement: Supplementary file 2 — Figure S2. The rarefaction curve plot for TDS1 (Red) and TDS2 (Blue) was depicted based on ACE, Chao1, richness, and Shannon diversity indices. The curves were generated based on 97% similarity threshold. [file MBO3-5-967-s002.tif]
